# Dipeptidyl Amino-Peptidase 3 (DPP3) as an Early Marker of Severity in a Patient Population with Cardiogenic Shock

**DOI:** 10.3390/diagnostics13071350

**Published:** 2023-04-04

**Authors:** Pasquale Innelli, Teresa Lopizzo, Giovanni Paternò, Noemi Bruno, Rosa Paola Radice, Pietro Bertini, Alberto Marabotti, Giampaolo Luzi, Eugenio Stabile, Aldo Di Fazio, Giuseppe Pittella, Gianluca Paternoster

**Affiliations:** 1 Acute Cardiac Care Unit, San Carlo Hospital, 85100 Potenza, Italy; 2 Clinical Pathology and Microbiology, San Carlo Hospital, 85100 Potenza, Italy; 3 Cardiac Intesive Care, San Camillo Forlanini, 00152 Rome, Italy; 4 Department of Science, Basilicata University, 85100 Potenza, Italy; 5 Department of Anesthesia and Critical Care Medicine, Azienda Ospedaliero Universitaria Pisana, 56126 Pisa, Italy; 6 Intensive Care Unit and Regional, ECMO Referral Centre, Azienda Ospedaliero-Universitaria Careggi, 50134 Florence, Italy; 7 Cardiac Surgery, San Carlo Hospital, 85100 Potenza, Italy; 8 Regional Complex Intercompany Institute of Legal Medicine, San Carlo Hospital, 85100 Potenza, Italy; 9 Cardiac Resuscitation, Cardiovascular Anesthesia and Intensive Care, San Carlo Hospital, 85100 Potenza, Italy

**Keywords:** DPP3, cardiogenic shock, mechanical ventilation, biomarker

## Abstract

Dipeptidyl amino-peptidase 3 (DPP3) is an aminopeptidase that is released into circulation upon cell death. DPP3 is involved in the degradation of angiotensins, enkephalines, and endomorphines. It has been shown that circulating DPP3 (cDPP3) plasma concentration increases in cardiogenic shock (CS) patients and correlates with high mortality risk. Cardiogenic shock is a life-threatening syndrome associated with organ hypoperfusion. One of the common causes of CS is acute myocardial infarction (AMI). This study aimed to investigate if cDPP3 levels are associated with CS severity and the need for ventilation in patients suffering from CS. Fifteen patients with CS were included in this study. Six patients were invasively ventilated. The values of cDPP3 were higher in ventilated patients than in non-ventilated patients at admission, 3 h, and 24 h after admission in the intensive care unit. Patients with pulmonary hypertension at admission also showed high cDPP3 values at all time points. Furthermore, high cDPP3 levels were associated with reduced stroke volume. Our results suggest that cDPP3 could predict CS progression and guide therapy escalation.

## 1. Introduction

### 1.1. Diseases of the Cardiovascular System 

Cardiovascular disease accounts for over two-thirds of total mortality worldwide and is an emerging serious health issue in middle- to low-income countries as well as in high-income countries [1]. Atherosclerotic cardiovascular disease is a collective term comprising of a group of disorders of the heart and blood vessels. These disorders are the largest cause of morbidity and death worldwide. Coronary heart disease, cerebrovascular disease and peripheral arterial disease are the most frequently occurring cardiovascular diseases. Early diagnosis of these disorders by means of better diagnostic tools is fundamental.

### 1.2. Cardiogenic Shock

Cardiogenic shock (CS) is a clinical condition in which ineffective cardiac output, caused by a primary cardiac disorder, results in inadequate tissue perfusion. The clinical presentation is typically characterized by persistent hypotension unresponsive to volume replacement and is accompanied by clinical features of end-organ hypoperfusion requiring intervention with pharmacological or mechanical support [2,3].

**Figure 1:**
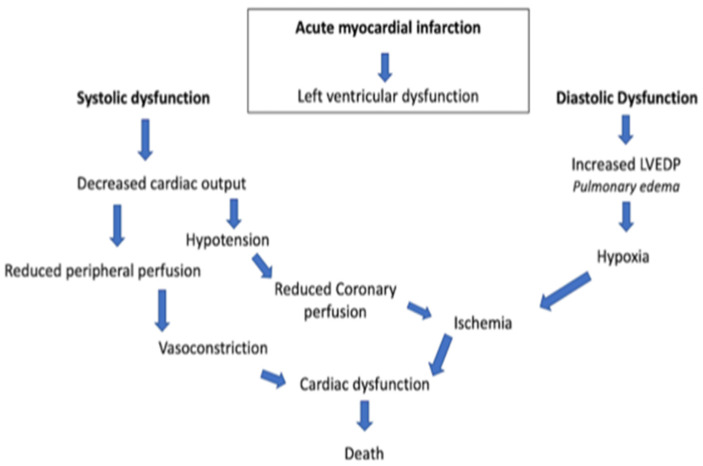
Acute myocardial infarction spiral. LVEDP, left ventricular end-diastolic pressure.

In general, there is a profound myocardial contractility depression resulting in a deleterious spiral of reduced cardiac output, low blood pressure, and further coronary ischemia (Figure 1). 

This classic paradigm also includes a lot of compensatory mechanisms, such as systemic vasoconstriction, that result in an ineffective stroke volume [4,5]. Consequently, CS and the downstream mechanisms induced thereby are strongly related to reduced end-organ perfusion with high risk of mortality and morbidity [6,7]. The management of CS frequently includes vasoactive drugs, circulatory and ventilatory support, and coronary revascularization in case of an acute myocardial infarction [8,9]. The management of CS is mostly based on clinicians’ experience rather than evidence-based recommendations since, for many treatments, either no adequately designed randomized clinical trials (RCT) exist or their results failed to show relevant beneficial effects. Hence, scarce advances have been achieved in the treatment of CS and patients’ outcomes remain poor [10]. In this context, particular attention is given to delineation of inclusion and exclusion criteria in RCTs in order to better understand the pathophysiology of patients that will and will not respond to medical treatment. Refractory cardiogenic shock refers to an ill-defined severity of CS not responding to standard therapies. In the pre-mechanical circulatory support (MCS) era, refractoriness to standard medical treatment led inevitably to death. The implementation of MCS opened new doors for the treatment of CS and modified the interpretation of “refractory” shock and its prognosis. However, the lack of a standard definition and nomenclature undermines research and clinical decision making in refractory CS.

### 1.3. Dipeptidyl Amino-Peptidase 3 in the Context of Cardiovascular Diseases

Dipeptidyl amino-peptidase 3 (DPP3) is an intracellular zinc-dependent enzyme [11] and is ubiquitously expressed in many cell types and tissues, including neutrophils, lungs, liver, kidney, and heart [12,13]. It has been reported that during cell death, intracellular DPP3 is released into circulation and, consequently, has been named circulating DPP3 (cDPP3) [14,15]. cDPP3 degrades angiotensins [16,17], enkephalines [17,18], and endorphines [19]. Therefore, it has been implicated in the modulation of many physiological processes, such as blood pressure regulation [16], inflammatory processes [13], and pain modulation [19]. The most notable DPP3 substrate is angiotensin II (Ang II), which serves as the primary effector molecule of the renin–angiotensin–aldosterone system (RAAS). The RAAS plays a vital role in regulating the cardiovascular system homeostasis, modulating blood pressure through the sympathetic system, glomerular filtration, releasing endogenous catecholamine and vasopressin, and stimulating vascular smooth muscle cells [20,21]. 

Increased blood levels of cDPP3 have been observed in critically ill patients suffering from septic, cardiogenic and haemorrhagic shock [22,23]. Furthermore, an association between high cDPP3 levels in the blood and high mortality in shocked patients in the ICU [22,23,24] has been shown. CS is a heterogenous syndrome associated with low cardiac output, organ hypoperfusion, and hypoxia, which leads to multi-organ failure and death [25,26]. The most common cause of cardiogenic shock remains acute myocardial infarction (AMI), representing 5–8% of patients with AMI [27]. Deniau et al. have shown that non-survivor CS patients had higher cDPP3 levels at all time points compared to survivors [24]. Furthermore, high cDPP3 levels in CS patients were associated with severe organ dysfunction. Consequently, cDPP3 levels at admission were predictive for 90-day mortality. Interestingly, a decrease in cDPP3 levels within 24 h of admission was associated with reduced 90-day mortality, decreased cardiovascular support, and renal function improvement [25]. 

Takagi et al. attempted to delineate a clinical role for cDPP3 and confirm the relationship between cDPP3 and clinical outcomes measured in patients with ST-elevation myocardial infarction (STEMI) and CS from the OptimaCC trial. The authors established the relationship between cDPP3 and the development of refractory CS, defined as shock non-responsive to inotropes and vasopressors with optimal cardiac filling pressures [22]. The authors have shown that cDPP3 levels are higher in patients that develop refractory CS within 72 h from admission to the ICU [22]. In addition, cDPP3 levels at admission could discriminate patients who did develop refractory shock from those who did not. Patients with high cDPP3 levels also exhibited lower cardiac index, low glomerular filtration rate, and higher Simplified Acute Physiology Score II (SAPS II) [22]. More importantly, CS patients with high cDPP3 levels at inclusion but decreased levels at 24 h showed a striking reduction in refractory shock and death [22]. 

Similar results have been observed in severe sepsis and septic shock patients. In this patient population, there is also a significant association between high cDPP3 blood levels upon ICU admission and 28-day mortality, high need for organ support, including prolonged need for vasopressor(s), mechanical ventilation and renal replacement therapy [28,29]. Interestingly, intravenous injection of DPP3 induced myocardial depression in healthy mice while inhibition of cDPP3 with Procizumab (a specific monoclonal antibody against cDPP3) normalized myocardial function in an acute heart failure mouse model, suggesting cDPP3 as a potential therapeutic target in patients with acute heart failure and shock. Pharmacological inhibition of cDPP3 by Procizumab also improved cardiac dysfunction and reduced mortality in septic rats [30]. 

### 1.4. Study Purpose

Helgestad et al. recently reported increased mechanical ventilation use in the CS population. Over 85% of the CS patients in the Danish Heart Failure data registry from 2010 to 2017 required mechanical ventilation by 2017 [31,32]. Despite treatment advances in CS therapy, 30-day mortality rates have been roughly unchanged over the past decades, remaining at about 50% [31,32]. Considering the syndromic nature of CS, advances in this field are likely to include many interventions.

One poorly researched aspect of patient care in CS is the management of respiratory support, despite the intimate cardiopulmonary relationship between positive pressure ventilation and ventricular function [33,34]. We, therefore, set out to investigate to what extent cDPP3 is associated with the need for mechanical ventilation and short-term outcomes in a CS patient population. Ultimately, the hypothesis-generating character of this study aims to instigate a further, biomarker-based investigation into the impact of mechanical ventilation on the cardiovascular system in CS.

## 2. Materials and Methods

### 2.1. Study Design and Population

We performed a single-center prospective study at San Carlo Azienda Ospedaliera Regionale in Italy, including fifteen patients; six patients were mechanically ventilated. The inclusion criteria for CS were systolic blood pressure (SBP) < 90 mmHg for > 30 min or supportive intervention to maintain SBP > 90 mmHg and evidence of end-organ damage (altered mental status, urine output (UO) < 30 mL/h, or cool extremities). Patients aged under 18 years or pregnant women were not included in the study. All patients were included from a non-surgical ICU. The study design included the recording of vital signs, arterial blood gas analysis, laboratory parameters, echocardiography at several time points, and medical history from the patient.

This study was carried out under the applicable rules concerning the review of research ethics committees and informed consent. All patients or legal representatives were informed about the details of this cohort study and could decline to participate. Blood samples were taken immediately upon admission to the ICU (baseline = adm), 3 h, 24 h, and one week (7 d). Patient discharge status and mortality were recorded on day 28 after ICU admission through medical examination if the patient was still hospitalized or through telephone interviews. Echocardiography was performed on admission to the ICU and after one week. 

### 2.2. Measurements

Ethylenediaminetetraacetic acid (EDTA)-anticoagulated blood for determination of cDPP3 concentration was sampled immediately upon admission to the ICU (adm); follow-up samples were acquired at 3 h, 24 h, and 1 week (7 d) post-admission. Blood samples were centrifuged at 2000× *g* at 4 °C for 15 min, after which plasma was stored at −80 °C. The blood samples for DPP3 measurement were shipped under controlled conditions to 4TEEN4 Pharmaceuticals GmbH. DPP3 was measured in EDTA plasma samples by 4TEEN4 Pharmaceuticals (Hennigsdorf, Germany) using the immunoluminometric assay sphingotest^®^ DPP3 (SphingoTec GmbH, Hennigsdorf, Germany) as described previously [15]. Based on the manufacturer’s instruction for use, the 97.5th percentile for sphingotest^®^ DPP3 in healthy adult subjects is 22 ng/mL (90% CI 18–34 ng/mL). 

### 2.3. Echocardiographic Measurements

The echocardiographic evaluation was performed using a Vivid I-q- GE-system machine according to Lang et al. At admission and one week post-admission, the following parameters were recorded: Left Ventricle End-Diastolic Diameter (EDD), Left ventricle End-Diastolic Volume (EDV), Left Ventricle Ejection Fraction (EF), Left Ventricle Stroke Volume normalized by BSA (SV), Left Atrial Volume (LAV), Right Ventricle Basal Linear Dimension (RDV1), Tricuspid Annular Longitudinal Excursion by M-mode (TAPSE), Right Ventricle Fractional Area Change (FAC) and Systolic Pulmonary Pressure (PAPs) [35]. EDD is acquired in the parasternal long-axis view carefully obtained perpendicular to the LV long axis and measured at the level of the mitral valve leaflet tips. 

EDV and ESV measurements are based on tracings of the blood–tissue interface in the apical four- and two-chamber views at end-diastolic and end-systolic time. The contour is closed at the mitral valve level by connecting the two opposite sections of the mitral ring with a straight line. LV length is defined as the distance between the middle of this line and the most distant point of the LV contour. EF is calculated from EDV and ESV estimates using the following formula: EF = (EDV − ESV)/EDV. SV is calculated by measuring the Doppler flow in the aortic valve. In the left ventricular outflow tract (LVOT), the following two measurements are performed:

(a) Diameter of the aortic annulus: This measurement is made in the parasternal long- axis view during systole, when the diameter is greatest (usually halfway through systole). Zoom in LVOT to improve the accuracy of the measurement was performed.
(b) Flow velocity in LVOT: Velocity is measured in apical four-chamber view (4C) or five-chamber view (5C) using pulsed-wave doppler with sample volume in the valve orifice. The VTI (Velocity Time Integral) is automatically calculated.

LAV measurement is based on tracings of the blood–tissue interface on apical four- and two-chamber views. At the mitral valve level, the contour is closed by connecting the two opposite sections of the mitral annulus within a straight line. Endocardial tracing should exclude atrial appendage and pulmonary veins. RVD1 measurement is based on the maximal transversal dimension in the basal one-third of RV inflow at end-diastole in the RV-focused view. TAPSE is measured between end-diastole and peak systole tricuspid annular longitudinal excursion by M-mode. FAC is measured in RV-focused apical four-chamber view using the following formula: RV FAC (%) = 100 × (End Diastolic Area (EDA) − End Systolic Area)/EDA. PAPs is calculated by applying the modified Bernoulli equation to the peak velocity of the tricuspid regurgitation jet: PAPs = 4v^2^_TR_ + RAP (right atrial pressure). RAP is estimated by inferior vena cava collapse [35].

### 2.4. Statistical Analyses

All continuous variables were assumed to be normally distributed and mean and standard deviation are given. Categorical variables are presented as counts and percentages. Categorical data were compared using Fisher’s-exact tests. Correlations between continuous variables were calculated using Pearson’s correlation. A two-sided *p*-value < 0.05 was regarded as statistically significant. Statistical analyses were performed using R, version 3.5.1 (R Foundation for Statistical Computing, Vienna, Austria). 

## 3. Results

Fifteen patients were included in the study. The mean age of the entire population was 69 ± 18.6 years and 46.7% were female. Six patients (40%) were mechanically ventilated. Six patients developed cardiogenic shock after STEMI. Of these six patients, two patients were mechanically ventilated. Five patients were admitted with congestive heart failure. Finally, four patients were diagnosed with valvular disease, cardiomyopathy, or Tako–Tsubo syndrome. These diagnoses are further referred to as others. Details of the clinical and biological parameters of the study population are summarized in Table 1. Two patients died seven days after admission to the intensive care unit (ICU). 

At admission, mean cDPP3 levels were 30.5 ± 20.6 ng/mL. The mean cDPP3 levels over time were 30.3 ± 21.1 ng/mL. At admission, high cDPP3 levels were associated with elevated CRP (*p* = 0.047). There was no significant association between cDPP3 levels and highly sensitive Troponin (hsTPN) and B-type natriuretic peptide (BNP) (*p* = 0.457 and *p* = 0.928).

**Figure 2:**
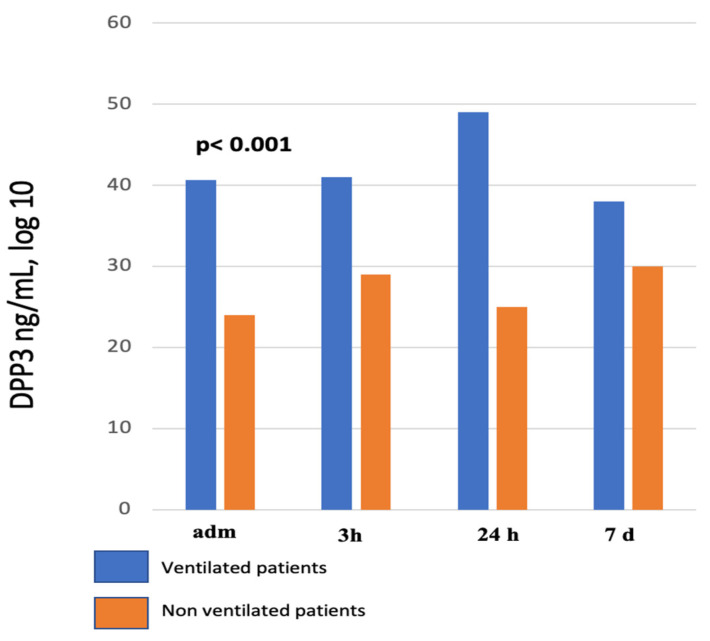
cDPP3 values within the first week in the ICU in ventilated and non-ventilated patients. At all timepoints, ventilated patients have significantly higher cDPP3 values compared to non-ventilated patients. Adm, admission to ICU.

At admission to the ICU, cDPP3 levels were higher in patients that were mechanically ventilated during their ICU stay (40.6 ± 30.9 ng/mL in ventilated patients verus 23.8 ± 4.6 ng/mL) in non-ventilated patients). In addition, cDPP3 levels during the first week in the ICU were significantly elevated in mechanically ventilated patients compared to non-ventilated patients (*p* < 0.001) (Figure 2; Table 2).

**Figure 3:**
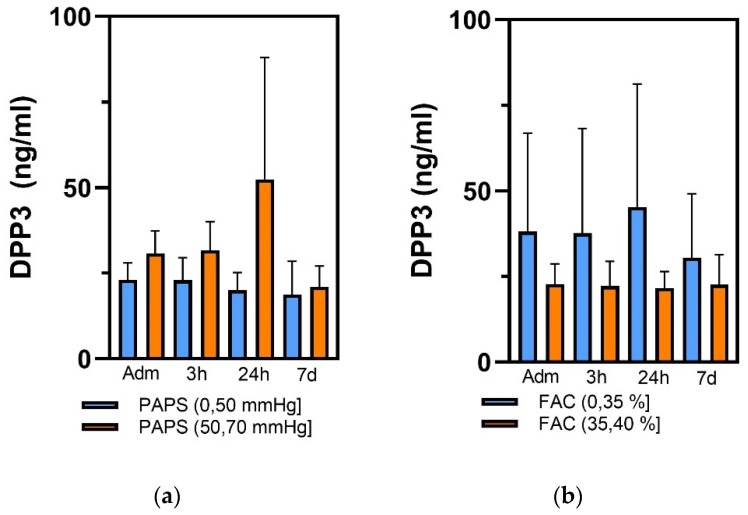
Graphs illustrating the distribution of cDPP3 levels in (**a**) patients with PAPS lower and higher than 50 mmHg and (**b**) patients with FAC lower and higher than 35% at time points at ICU admission (adm) and 3 h, 24 h and 7 days (7 d) post ICU admission.

No significant correlation in echocardiographic data between the ventilated and non-ventilated patients could be observed (Table 3). High cDPP3 values during the first week in the ICU were significantly associated with reduced FAC (below 35%) (*p* = 0.008) and increased PAPS (above 50 mmHg) (*p* = 0.006) at admission (Figure 3). Although cDPP3 levels at admission did not discriminate based on stroke volume, patients with decreased stroke volume (below 28 mL/m^2^) showed a tendency toward high cDPP3 levels at 3 and 24 h post-admission. Finally, cDPP3 at admission was elevated in non-survivors (63.8 ± 54.2 ng/mL) compared to survivors (25.4 ± 6.3 ng/mL) and remained significantly elevated in non-survivors during the ICU stay (*p* < 0.001).

## 4. Discussion

During the early clinical course, it is challenging to predict which patients, who have had an acute myocardial infarction, will eventually develop refractory CS. To address this problem, researchers have been exploring the use of biomarkers for early identification of those at risk of developing refractory CS in order to perform earlier interventions involving MCS or early patient transfer to higher levels of care (shock centers) in the event that MCS is not immediately available.

Early identification of patients at risk could lead to improved treatment strategies, reduced length of stay in the ICU and consequently better outcomes. N-terminal pro-B-type natriuretic peptide (NT-proBNP) is considered one of the most promising biomarkers for the prediction of refractory CS. This biomarker has been found to be associated with an increased risk of refractory CS in patients with acute myocardial infarction. Other biomarkers that have been studied include troponin, C-reactive protein (CRP), and brain natriuretic peptide (BNP).

In our small study population, we were able to demonstrate that cDPP3 levels in the blood correlate with the severity of CS and follow the disease progression. According to our findings, cDPP3 values are particularly high in more severe CS patients who require orotracheal intubation and mechanical ventilation. This data is consistent with previous studies in septic shock patients, which demonstrated a detrimental association of cDPP3 values and organ function [28]. In septic shock patients, high admission cDPP3 values were observed in patients with high demand for vasopressors and, most interestingly, were significantly associated with need and duration of invasive mechanical ventilation. In addition, high cDPP3 levels in CS are also correlated with an increase in mortality.

Hypoxemia, increased difficulty of breathing, and decreased level of consciousness may require airway orotracheal intubation with mechanical ventilation, which is usually linked to systemic hemodynamic instability and increased mortality. Acute respiratory failure (ARF) is well studied in the setting of non-cardiogenic shock patients that present with pneumonia or adult respiratory distress syndrome. However, data to support a particular mode of ventilation or physiological targets for oxygenation or ventilation in CS is scarce. In addition, this topic is not often studied in main clinical trials. In a recent systematic review and meta-analysis [9] on the features of RV impairment and pulmonary hypertension (PH) in COVID-19 patients under mechanical ventilation and its impact on mortality, we found alterations in RV function or PH dimensions in patients with COVID-19 undergoing respiratory support. This effect seems to play a major role in determining mortality rate in this patient population. These findings could be applicable to CS patients and, therefore, clinicians must be mindful of the interactions between positive pressure ventilation on left and right ventricular hemodynamics as well as the optimal agents for analgesia and sedation. Considering the syndromic nature of CS, an improved definition of CS severity and selection of proper inclusion criteria for RCTs including novel biomarkers, hemodynamics and metabolic parameters could lead to a paradigm shift in the way we approach medical care, providing new, more potent and cost-effective treatments than current therapies. Furthermore, it could boost the development of new diagnostic tools, allowing for earlier detection of hemodynamic derangement [36,37]. Finally, the knowledge of the molecular mechanisms underlying CS could lead to targeted treatment strategies and open doors to personalized medicine, allowing clinicians to tailor treatments to individual patients based on their unique phenotype.

## 5. Conclusions

The use of cDPP3 could be beneficial in helping clinicians make decisions on how to approach cardiogenic shock, such as recommendation of rapid transfer to a tertiary care center or the use of an intra-aortic balloon pump for high cDPP3 patients. Additionally, cDPP3 could be used to monitor a patient’s progress and help guide therapeutic decisions. Decreasing cDPP3 levels could lead to a recommendion for a less aggressive approach. Furthermore, cDPP3 together with other clinical and biological parameters could be used as a population-enrichment strategy in CS trials [38]. Finally, randomized clinical trials should clarify whether pharmacological inhibition of cDPP3 could be a novel therapeutic approach to improve hemodynamics and cardiovascular prognosis in CS patients.

### Strengths and Limitations

The major limitations of this observational study are the small sample size of the studied population and its etiologic heterogeneity. We mainly showed associations between cDPP3 levels and the severity of cardiogenic shock; we did not provide molecular insights into the underlying mechanisms downstream of cDPP3 release in the circulation and dedicated experimental studies are necessary to further elucidate these pathophysiological pathways. It is essential that the hypothesis-generating nature of this study be emphasized despite the fact that the results are encouraging. The results shown in this study need to be validated in a prospective study, including a more significant number of CS patients with a special focus on the predictive value of cDPP3 levels in ventilated and non-ventilated patients with cardiogenic shock.

## Figures and Tables

**Table 1:** Main characteristics of the whole study population, divided by ventilated and non-ventilated patients.

Baseline Variables	All Patients (*n* = 15) (%)	Ventilated (*n* = 6 (40%))	Non-Ventilated (*n* = 9 (60%))	*p*-Value
Gender (F)	7 (46.7%)	2 (33%)	5 (55.6%)	0.002
Age (years)	69.0 ± 18.6	61.0 ± 8.5	67.4 ± 18.7	0.329
SBP (mmHg)	79.5± 8.5	70.1 ± 9.4	87 ± 9.1	0.038
Hb (g/dL)	12.0 ± 2.0	10.5 ± 1.8	12.1 ± 2.1	0.128
BNP (mmol/L)	971,6	989.0 ± 217.0	1036.7 ± 325.0	0.750
HS Trop (mmol/L)	37,692.9 ± 86,529.8	57,867.0 ± 109,964.0	4334.0 ± 7993.0	0.659
CRP (mmol/L)	31.8 ± 60.1	99.5 ± 79.8	27.5 ± 53.3	0.047
STEMI, *n* (%)	6 (40%)	2 (33.3%)	4 (44.4%)	0.0008
CHF, *n* (%)	5 (33%)	2 (33.3%)	3 (33.3%)	0.0004
Other, *n* (%)	4 (26.7%)	2 (33.3%)	2 (22%)	0.0004
Diabetes, *n* (%)	4 (26.7%)	3 (50%)	1 (11%)	0.177
Hypertension, *n* (%)	7 (46.7%)	4 (66.7%)	3 (33.3%)	0.061
Hypercholesterolemia, *n* (%)	10 (66.7%)	6 (100%)	4 (44.4%)	0.218
Chronic Kidney Disease, *n* (%)	3 (20%)	0	3 (33.3%)	<0.000001
Valvular Regurgitation, *n* (%)	6 (40%)	1 (16.7%)	5 (55.6%)	0.00008

SBP: Systolic Blood Pressure; Hb: Hemoglobin BNP: Brain Natriuretic Peptide; HsTrop: Hight Sensitivity Troponin; CRP: C-Reactive Protein; STEMI: ST-segment elevation myocardial infarction; CHF: Chronic Heart Failure.

**Table 2:** cDPP3 values (in ng/mL) in ventilated and non-ventilated patients. The data are reported as mean ± standard deviation.

Patients	Admission	3 h	24 h	7 d
Ventilated (*n* = 6)	132.4 ± 73.0	130.1 ± 67.1	109.4 ± 73.7	86.0 ± 71.1
Non-ventilated (*n* = 9)	93.6 ± 93.1	93.5 ± 93.3	60.0 ± 52.9	108.7 ± 111

**Table 3:** Echocardiographic data at admission. The data are reported as mean ± standard deviation.

ECHO Parameters	Entire Cohort (*n* = 15) (%)	Ventilated (*n* = 6 (40%))	Not-Ventilated (*n* = 9 (60%))	*p*-Value
EDV (mL)	91.7 ± 37.1	92.2 ± 36.5	91.2 ± 43.3	0.978
EDD (mL)	48.5 ± 6.5	45.7 ± 4.3	51.2 ± 7.8	0.346
EF (%)	36.1 ± 12.3	29.6 ± 7.7	42.5 ± 12.1	0.247
SV (mL/m^2^)	30.2 ± 14.3	22.7 ± 4.2	37.7 ± 17.6	0.228
LAV (mL)	60.4 ± 22.8	47.5 ± 18.1	77.6 ± 17.1	0.233
RVD1 (mm)	36.7 ± 11.1	38.7 ± 10.8	35.6 ± 12.1	0.711
TAPSE (mm)	13.5 ± 5.7	15.2 ± 6.1	19.1 ± 12.7	0.552
FAC (%)	29.9 ± 7.5	30.7 ± 10.7	36.2 ± 2.6	0.847
PAPS (mmHg)	42.8 ± 13.5	51.0 ± 17.3	52.0 ± 11.3	0.259

EEDV End Diastolic volume; EDD End Diastolic Diameter; EF: Ejection Fraction; SV: Stroke Volume; LAV: Left atrial Volume; RVD1: Right Ventricle Basal Linear Dimension; TAPSE Tricuspid Annular Longitudinal Excursion; FAC: Right Ventricle Fractional Area Change; PAPs Systolic Pulmonary Pressure.

## Data Availability

Data are not available due to privacy restrictions. However, the authors are available to evaluate any requests.
